# Longitudinal changes in ventricular size and function are associated with death and transplantation late after the Fontan operation

**DOI:** 10.1186/s12968-022-00884-y

**Published:** 2022-11-14

**Authors:** Sunil J. Ghelani, Minmin Lu, Lynn A. Sleeper, Ashwin Prakash, Daniel A. Castellanos, Nicole St. Clair, Andrew J. Powell, Rahul H. Rathod

**Affiliations:** 1 Department of Cardiology, Boston Children’s Hospital, 300 Longwood Ave, Boston, MA 02115 USA; 2 Department of Pediatrics, Harvard Medical School, Boston, MA USA

**Keywords:** Magnetic resonance imaging, Congenital heart disease, Fontan operation

## Abstract

**Background:**

Cross-sectional studies have reported that ventricular dilation and dysfunction are associated with adverse clinical outcome in Fontan patients; however, longitudinal changes and their relationship with outcome are not known.

**Methods:**

This was a single-center retrospective analysis of Fontan patients with at least 2 cardiovascular magnetic resonance (CMR) scans without intervening interventions. Serial measures of end-diastolic volume index (EDVI), end-systolic volume index (ESVI), ejection fraction (EF), indexed mass (mass_*i*_), mass-to-volume ratio, and end-systolic wall stress (ESWS) were used to estimate within-patient change over time. Changes were compared for those with and without a composite outcome (death, heart transplant, or transplant listing) as well as between patients with left (LV) and right ventricular (RV) dominance.

**Results:**

Data from 156 patients were analyzed with a mean age at 1st CMR of 17.8 ± 9.6 years. 490 CMRs were included with median of 3 CMRs/patient (range 2–9). On regression analysis with mixed effects models, volumes and ESWS increased, while mass, mass-to-volume ratio, and EF decreased over time. With a median follow-up of 10.2 years, 14% met the composite outcome. Those with the composite outcome had a greater increase in EDVI compared to those without (4.7 vs. 0.8 ml/BSA^1.3^/year). Compared with LV dominance, RV dominance was associated with a greater increase in ESVI (1.4 vs. 0.5 ml/BSA^1.3^/year), a greater decrease in EF (− 0.61%/year vs. − 0.24%/year), and a higher rate of the composite outcome (21% vs. 8%).

**Conclusions:**

Ventricles in the Fontan circulation exhibit a steady decline in performance with an increase in EDVI, ESVI, and ESWS, and decrease in EF, mass index, and mass-to-volume ratio. Those with death or need for heart transplantation have a faster increase in EDVI. Patients with rapid increase in EDVI (> 5 ml/BSA^1.3^/year) may be at a higher risk of adverse outcomes and may benefit from closer surveillance. RV dominance is associated with worse clinical outcomes and remodeling compared to LV dominance.

**Supplementary Information:**

The online version contains supplementary material available at 10.1186/s12968-022-00884-y.

## Background

Originally performed in the late 1960s, the Fontan operation provides a pathway to survival for children with single ventricle heart defects [1, 2]. In the current era, the Fontan circulation is typically achieved via staged operations which sequentially alleviate cyanosis and ventricular volume load [3]. The number of patients with a Fontan circulation worldwide is estimated to be greater than 70,000 and is expected to double over the next 20 years [4]. Morbidity and early mortality are common in this population with estimated 20-year survival of 61–85%, and a risk of late mortality of approximately 2.1% per year [5–9]. Risk-stratification in the Fontan population is important to identify patients who might benefit from closer follow-up, medical therapy, and catheter-based or surgical interventions. Routine surveillance testing recommendations include echocardiograms every year and cardiovascular magnetic resonance imaging (CMR) every 2–3 years [4]. Prior cross-sectional studies have established ventricular dilation, dysfunction, and the presence of late gadolinium enhancement (LGE) on CMR as risk factors for adverse clinical outcomes [9–12].

The aim of current study was to analyze serial CMRs without interim interventions from a single large-volume center to characterize changes in ventricular size and function over time. The study hypothesized that significant temporal trends will exist in ventricular parameters and that the rate of change of these parameters will be associated with death or need for heart transplantation.

## Methods

### Subjects

This was a single-center retrospective analysis of all patients who had undergone a Fontan operation and had at least 2 CMR examinations without catheter-based or surgical cardiac interventions between the first and last CMR. Studies in which ventricular volumes could not be reliably measured due to artifact were excluded. Demographic and clinical data were abstracted from medical records. The study’s composite outcome was defined as death from any cause, heart transplantation, or listing for heart transplantation.

### CMR protocol

CMR examinations were performed on 1.5 T CMR scanners (Philips Healthcare, Best, the Netherlands and General Electric Healthcare, Chicago, Illinois, USA). Details of the study protocol in our lab have been previously published [13]. Ventricular measurements were made on a short-axis cine stack of electrocardiographic (ECG)-gated, breath-hold balanced steady-state free precession images. The spatial resolution was 1.7–2 mm by 1.7–2 mm with a slice thickness of 8 mm. For each slice, 30 phases were reconstructed with a temporal resolution of 30–40 ms. Ventricular mass and volumes were measured by manually tracing endocardial and epicardial borders at end-diastole (maximal volume) and end-systole (minimum volume). When two ventricles contributed to the systemic circulation, the ventricular volumes were added. Ventricular dominance was defined as left (LV) or right (RV) based on the sole or larger of the ventricles contributing to the systemic circulation. The following indices were recorded: end-diastolic volume index (EDVI), end-systolic volume index (ESVI), ejection fraction (EF), ventricular mass index and mass-to-volume ratio. Indexing was performed for mass and volume measurements to BSA^1.3^ [14]. Ventricular end-systolic wall stress (ESWS) was calculated using the mean blood pressure, and ventricular and myocardial volumes as previously described [15]. The degree of atrioventricular valve regurgitation (AVVR) and semilunar valve regurgitation was graded as more than mild if the regurgitation fraction was > 20%. When two valves were present, regurgitation fractions were combined. CMR measurements were obtained from imaging reports. Ventricular volumes were remeasured in a randomly selected subset of 20 patients by a single observer to assess reproducibility.

### Statistical analysis

Descriptive statistics are presented as mean ± standard deviation (SD) for continuous variables except when indicated. Categorical data are described as frequency with percentage. Where mean comparisons among independent groups are presented, a Student’s t-test or ANOVA was performed, and for comparisons of categorical variables, a Fisher exact test was performed. To evaluate whether percentage change in CMR parameters differed from zero, a one sample t-test was used. Longitudinal changes in CMR parameters were estimated using two methods. First as an annualized change per individual patient defined as (last study − first study)/number of years between the studies, and second by using a mixed effects model (random intercept for each subject, fixed time effect) to assess rates of change ± standard error (SE) over time in the overall cohort. No CMRs obtained after the occurrence of listing for transplant were included in analyses. Rates of changes in CMR parameters were compared between those with and without the composite outcome using a test of interaction. Comparisons were also performed between patients with LV and RV dominant anatomy, regardless of clinical outcome. A p-value < 0.05 was defined as statistically significant. Interobserver reliability was assessed for ventricular volumes by using a two-way mixed effects model to estimate the intraclass correlation coefficient (ICC), percent error, and coefficient of variability, and is visually depicted in the form of Bland–Altman plots. Analyses were performed using SAS (version 9.4, SAS Institute, Inc., Cary, North Carolina, USA) including SAS Proc Mixed, and R (version 4.0.3, R Foundation for Statistical Computing, Vienna, Austria).

## Results

Analysis included 156 Fontan patients and a total of 490 CMRs. Median date of CMR was 4/11/2010 (Range 3/11/1999–7/14/17; IQR 1/22/2007–5/15/2014). The median number of CMRs per patient was 3 (range 2–9). The mean age at the first CMR was 17.8 ± 9.6 years, median follow-up time after the first CMR was 10.2 years (IQR 6.1–14.5 years), and median follow-up time after the last CMR was 3.8 (IQR 2.5–6.5 years). The median time between 2 CMRs was 5 years (IQR 2.3–8.7 years). Median number of CMRs in patients with the composite outcome was 3 (IQR 2–3) compared to 2 (IQR 2–4; p = 0.219) in those without the outcome. The primary underlying cardiac diagnosis was tricuspid atresia in 21%, hypoplastic left heart syndrome in 20%, double inlet LV in 19%, double outlet RV in 12%, unbalanced common atrioventricular canal defect in 9%, small right heart structures including pulmonary atresia with intact ventricular septum in 8%, and other rare forms of functional single ventricles in the remainder. Ventricular dominance was LV in 53% and RV in 47%. 135 (87%) ventricles were D looped, 17 (11%) were L lopped, and 4 were classified as unknown.

Table 1 shows the results of a mixed effects model to assess rates of change over time in the overall cohort. Mean EDVI and ESVI increased; mass index, mass-to-volume ratio, and EF decreased over time, and ESWS rose significantly. Inferences were unchanged after exclusion of outliers (Additional file 1: Table 1A and 1B). There were no significant interactions between age-group at first CMR and longitudinal changes in CMR measurements (Additional file 1: Table 6). When age at CMR was controlled for as a continuous variable, inferences were unchanged; although, the age-adjusted annual increase in EDVI was higher at 1.63 ml/BSA^1.3^ and annual decrease in mass index was higher at 1.70 g/BSA^1.3^. More than mild AVVR was present on the initial CMR in 14% of the cohort and more than mild semilunar valve regurgitation in 3%. Their presence was not associated with longitudinal changes in CMR variables although the analysis may have been underpowered due to small sample size (Additional file 1: Table 7). In 39 of the 156 patients (25%), the smaller ventricle was at least 20% of the combined ventricular volume on the first CMR. Of these 39 patients, 18 (46%) were LV dominant and 21 (54%) were RV dominant. Patients without a significant sized second ventricle showed a faster increase in ventricular end-diastolic volume (1.41 ± 0.28 ml/BSA^1.3^) as compared to those with (0.01 ± 0.43 ml/BSA^1.3^, p = 0.007; Additional file 1: Table 8).

**Table 1:** Model-based Estimates of Annual Change in Cardiovascular Magnetic Resonance (CMR) Parameters in 156 Patients (median 3 CMRs per patient, range 2–9)

Variables	N of CMRs	Estimated Annual Change (SE)	p-value
EDVI, ml/BSA^1.3^/year	490	0.99 (0.24)	**< 0.001**
ESVI, ml/BSA^1.3^/year	490	0.93 (0.20)	**< 0.001**
Mass index, g/BSA^1.3^/year	481	− 1.23 (0.16)	**< 0.001**
EF, %/year	490	− 0.41 (0.07)	**< 0.001**
Mass-to-volume ratio /year	481	− 0.02 (0.002)	**< 0.001**
ESWS, kPa/year	417	0.27 (0.08)	**< 0.001**

Bold indicates p-value < 0.05

*EDVI* end-diastolic volume index, *EF* ejection fraction, *ESVI* end-systolic volume index, *ESWS* end-systolic wall stress

**Fig. 1:**
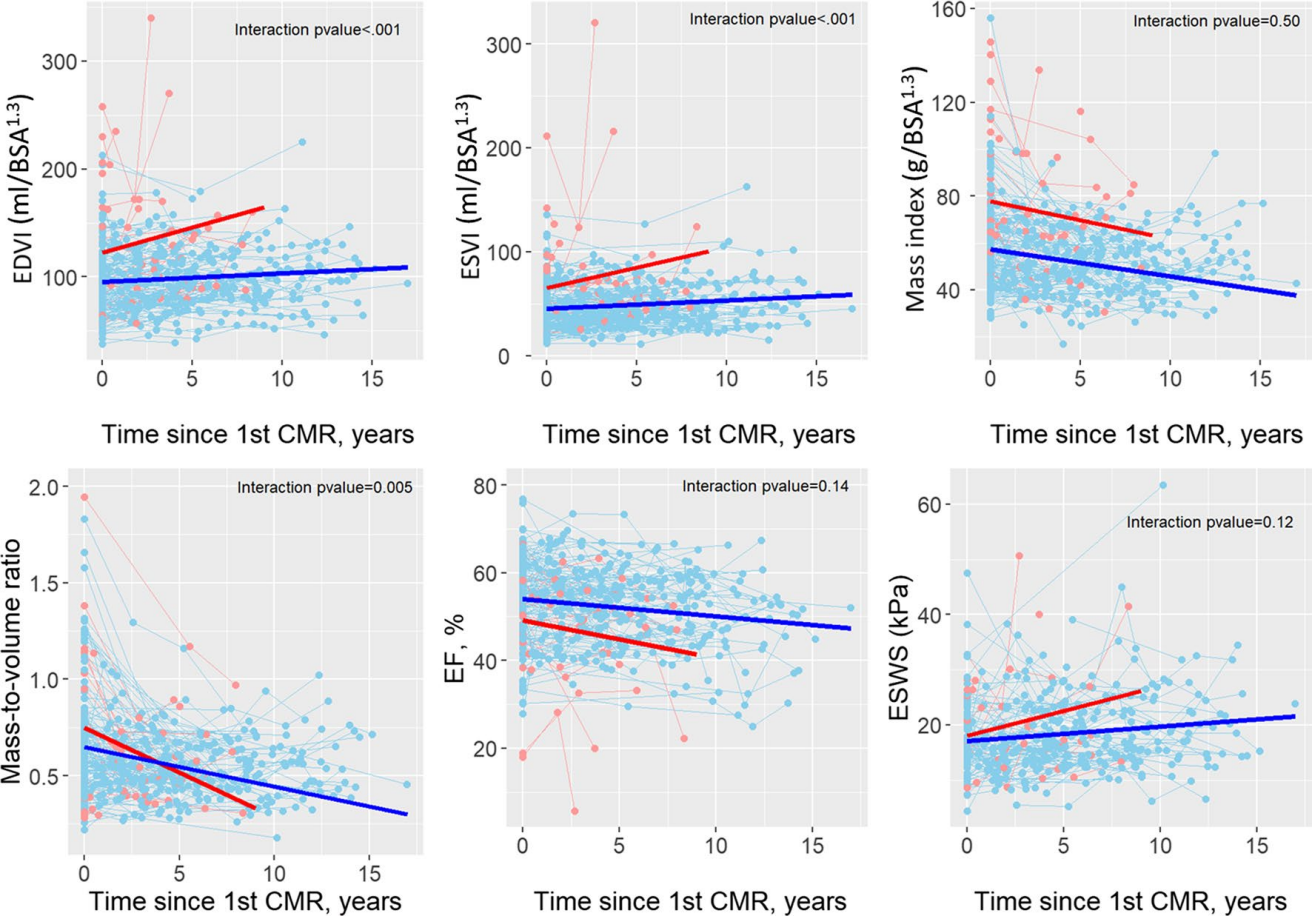
Estimated change in cardiovascular magnetic resonance (CMR) parameters over time, according to presence vs. absence of clinical outcome. Red line and points denote clinical outcome and blue line and points denote no clinical outcome. A significant change over time was observed in both groups for all CMR parameters. However, those with the composite outcome (red line) had a greater increase in end-diastolic volume index (EDVI) and end-systolic volume index (ESVI) and a greater decline in mass-to-volume ratio over time as compared to those without (blue line). There was no difference in the change over time in mass index, ejection fraction (EF), or end-systolic wall stress (ESWS) for those who did vs. did not experience the composite outcome

Table 2 describes the CMR parameters at the first and last CMR, and the annualized change by composite outcome status. The composite outcome was observed in 22 (14%) patients (17 deaths and 5 transplants or listings for transplant). Patients with the composite outcome had higher mean EDVI, and a lower mass index and EF on the first CMR. Annualized change over time was also associated with outcome: those with the composite outcome had a higher increase in EDVI, ESVI, and ESWS; and a decrease in mass index, mass-to-volume ratio, and EF. Table 3 shows results of mixed models regression with interaction of time and composite outcome status for CMR measurements. It shows that while both groups had a significant change over time, the magnitude of change differed according to outcome status only for EDVI, ESVI, and mass-to-volume ratio. Inferences were unchanged on repeating this analysis after exclusion of extreme outliers (outside of IQR × 3; Additional file 1: Table 3A). Analysis after exclusion of measurements outside of IQR × 1.5, demonstrated that magnitude of change differed according to the outcome status only for EDVI (Additional file 1: Table 3B). Figure 1 depicts the model-based estimates of change over time in CMR parameters for patients with and without the composite outcome.

**Table 2:** CMR parameters and annualized change by composite outcome status

Parameter	Overall (n = 156)	Composite outcome		p value
		Yes (n = 22)	No (n = 134)	
Follow-up time, years, Median (IQR)	10.2 (6.1,14.5)	6.2 (3.9, 8.4)	10.8 (6.9, 15.3)	**< 0.001**
Male	100 (64.1%)	14 (63.6%)	86 (64.2%)	0.96
Ventricular dominance				**0.03**
Left ventricle	83 (53.2%)	7 (31.8%)	76 (56.7%)	
Right ventricle	73 (46.8%)	15 (68.2%)	58 (43.3%)	
At the first CMR				
Age at CMR, years	17.8 ± 9.6	22.0 ± 13.5	17.1 ± 8.6	0.11
BSA, m^2^	1.47 ± 0.41	1.57 ± 0.53	1.46 ± 0.39	0.34
EDVI, ml/BSA^1.3^	98.3 ± 37.5	120.9 ± 58.3	94.6 ± 31.7	**0.05**
ESVI, ml/BSA^1.3^	47.7 ± 26.8	63.9 ± 43.8	45.0 ± 22.0	0.06
Mass index, g/BSA^1.3^	62.0 ± 23.1	80.6 ± 32.5	58.8 ± 19.6	**0.005**
EF, %	53.3 ± 10.0	49.5 ± 12.4	54.0 ± 9.5	0.05
Mass-to-volume ratio	0.69 ± 0.31	0.79 ± 0.43	0.67 ± 0.29	0.23
ESWS, kPa	17.7 ± 6.7	20.5 ± 7.5	17.4 ± 6.6	0.17
At the last CMR	n = 155^b^	n = 21	n = 134	
Age at CMR, years	23.7 ± 10.6	26.7 ± 14.5	23.3 ± 9.9	0.30
Time between 2 CMRs, Median (IQR)	5.0 (2.3, 8.7)	4.4 (2.0, 5.9)	5.1 (2.3, 9.0)	**0.04**
BSA, m^2^	1.69 ± 0.37	1.72 ± 0.46	1.68 ± 0.36	0.71
EDVI, ml/BSA^1.3^	106.3 ± 39.5	146.3 ± 69.9	100.0 ± 28.0	**0.007**
ESVI, ml/BSA^1.3^	54.8 ± 34.5	88.7 ± 68.3	49.5 ± 21.6	**0.02**
Mass indx, g/BSA^1.3^	54.2 ± 18.0	73.5 ± 26.5	51.2 ± 14.2	**0.001**
EF, %	50.9 ± 9.9	43.8 ± 13.8	52.0 ± 8.7	**0.01**
Mass-to-volume ratio	0.54 ± 0.16	0.56 ± 0.24	0.53 ± 0.14	0.58
ESWS, kPa	18.8 ± 7.8	22.4 ± 10.9	18.3 ± 7.1	0.11
Annual change in CMR (last-first)/(years between last and first)				
EDVI, ml/BSA^1.3^/year, Median (IQR)	0.93 (− 0.97, 3.20)^a^	4.68 (1.11, 8.79)^a^	0.70 (− 1.40, 2.64)^a^	**0.003**
ESVI, ml/BSA^1.3^/year, Median (IQR)	0.84 (− 0.72, 2.51)^a^	3.62 (0.91, 9.60)^a^	0.70 (− 0.76, 1.99)^a^	**< 0.001**
Mass index, g/BSA^1.3^/year, Median (IQR)	− 1.06 (− 3.12, 0.71)^a^	− 4.49 (− 5.30, 0.36)	− 0.92 (− 2.21, 0.71)^a^	**0.04**
EF, %/year, Median (IQR)	− 0.35 (− 1.25, 0.41)^a^	− 0.99 (− 5.12, 0.29)^a^	− 0.30 (− 1.17, 0.41)^a^	**0.03**
Mass-to-volume ratio, /year, Median (IQR)	− 0.02 (− 0.05, 0.01)^a^	− 0.06 (− 0.10, − 0.03)^a^	− 0.01 (− 0.04, 0.01)^a^	**0.006**
ESWS, kPa/year, Median (IQR)	0.30 (− 0.59, 1.23)	3.12 (1.55, 4.97)^a^	0.17 (− 0.73, 0.81)	**0.002**

Bold indicates p-value < 0.05

^a^Indicates that the change is significantly different from 0

^b^One patient had transplant listing prior to their last CMR  *BSA*, body surface area; *CMR*, cardiovascular magnetic resonance; *LV*, left ventricle; *RV*, right ventricle/right ventricular

**Table 3:** Estimates of annual change in CMR parameters by composite outcome status

Variable	Estimated annual change (SE)^a^		p-value^b^
	Composite outcome	No composite outcome	
EDVI, ml/BSA^1.3^/year	4.66 (1.01)	0.82 (0.24)	**< 0.001**
ESVI, ml/BSA^1.3^/year	3.90 (0.84)	0.79 (0.20)	**< 0.001**
Mass index, g/BSA^1.3^/year	− 1.62 (0.68)	− 1.14 (0.16)	0.50
EF %/year	− 0.86 (0.31)	− 0.39 (0.07)	0.14
Mass-to-volume ratio, /year	− 0.05 (0.009)	− 0.02 (0.002)	**0.005**
ESWS, kPa/year	0.88 (0.39)	0.26 (0.08)	0.12

Bold indicates p-value < 0.05

^a^All estimates significantly differed from zero, denoting significant change over time

^b^A p-value less than 0.05 indicates that the slopes of the two groups differ

More patients experienced the composite outcome in the RV dominant group as compared with the LV dominant group (21% vs. 8%, p = 0.03). Table 4 describes the CMR parameters at the first CMR, at the last CMR, and the annualized change by LV dominant vs. RV dominant group. On the first CMR, the RV dominant group had larger mean indexed volumes, ESWS, and lower mass-to-volume ratio. There was no difference in EF for RV vs. LV dominant patients on the first CMR, but the RV dominant group had a significant reduction in EF over time; hence had a lower EF on the last CMR. The estimated annual rate of change for the whole cohort using mixed models with interaction of time and dominant ventricle are presented in Table 5. There was a greater increase in ESVI and a greater decline in EF in the RV dominant group compared with the changes observed in the LV dominant group. Estimated annual change in the remaining CMR parameters was not associated with ventricular dominance. Interobserver reliability analysis for ventricular end-diastolic volume demonstrated a high level of agreement (ICC 0.99, Coefficient of variability 1.83%; Additional file 1: Table 9 and Fig. 4).

**Table 4:** CMR characteristics and longitudinal changes by ventricular dominance

Variable	Dominant ventricle		p-value
	LV (n = 83)	RV (n = 73)	
Follow up time since first CMR, years Median (IQR)	10.7 (6.4, 14.5)	9.2 (5.8, 14.3)	0.13
Composite outcome			**0.03**
Listing/Death/Transplant	7 (8.4%)	15 (20.5%)	
Free of event	76 (91.6%)	58 (79.5%)	
Male	51 (61.4%)	49 (67.1%)	0.461
At the first CMR			
Age at CMR, years	19.7 ± 10.0	15.6 ± 8.7	**0.008**
Height, cm	155 ± 20	149 ± 24	0.10
Weight, kg	54.5 ± 19.9	50.0 ± 23.1	0.19
BSA, m^2^	1.52 ± 0.37	1.42 ± 0.44	0.14
EDVI, ml/BSA^1.3^	88.9 ± 28.6	109.0 ± 43.3	**< 0.001**
ESVI, ml/BSA^1.3^	42.3 ± 20.4	53.8 ± 31.7	**0.009**
Mass_*i*_, g/BSA^1.3^	63.3 ± 23.9	60.5 ± 22.3	0.46
EF, %	53.9 ± 10.3	52.7 ± 9.6	0.42
Mass-to-volume ratio	0.76 ± 0.31	0.62 ± 0.30	**0.005**
ESWS, kPa	16.0 ± 5.3	19.7 ± 7.7	**0.006**
At the last CMR	n = 83^b^	n = 72	
Age at CMR, years	25.7 ± 11.0	21.5 ± 9.8	**0.013**
Time between 2 CMRs, years	6.0 ± 4.1	5.7 ± 4.0	0.68
Median (IQR)	5.5 (2.0, 8.6)	4.8 (2.6, 8.7)	0.62
BSA, m^2^	1.70 ± 0.34	1.67 ± 0.41	0.59
EDVI, ml/BSA^1.3^	96.7 ± 25.4	117.4 ± 49.1	**0.002**
ESVI, ml/BSA^1.3^	46.4 ± 18.7	64.4 ± 44.8	**0.002**
Mass index, g/BSA^1.3^	54.5 ± 16.3	53.9 ± 20.0	0.86
EF, %	53.2 ± 8.3	48.3 ± 11.0	**0.002**
Mass-to-volume ratio	0.58 ± 0.15	0.49 ± 0.16	**< 0.001**
ESWS, kPa	16.6 ± 7.1	21.4 ± 8.0	**< 0.001**
Annual Change in CMR (last-first)/(years between last and first)			
EDVI, ml/BSA^1.3^/year Median (IQR)	1.09 (− 0.69, 3.45)^a^	0.71 (− 1.61, 2.75)	0.48
ESVI, ml/BSA^1.3^/year Median (IQR)	0.69 (− 0.70, 1.99)^a^	1.22 (− 0.74, 2.66)^a^	0.24
Mass index, g/BSA^1.3^/year Median (IQR)	− 1.18 (− 2.98, 0.55)^a^	− 0.93 (− 3.47, 1.05)^a^	0.70
EF, %/year Median (IQR)	− 0.12 (− 0.98, 0.66)	− 0.62 (− 2.21, 0.27)^a^	**0.02**
Mass-to-volume ratio, /year Median (IQR)	− 0.02 (− 0.05, 0.01)^a^	− 0.01 (− 0.03, 0.01)^a^	0.24
ESWS, kPa/year Median (IQR)	0.31 (− 0.37, 0.78)	0.30 (− 0.78, 1.40)	0.81

Bold indicates p-value < 0.05

^a^Indicates that the change is significantly different from 0

^b^One patient had transplant listing prior to their last CMR

**Table 5:** Estimates of annual change in CMR parameters by ventricular dominance

Variable	Estimated Annual Change (SE)^a^		p-value^b^
	LV dominant	RV dominant	
EDVI, ml/BSA^1.3^/year	0.73 (0.32)	1.27 (0.35)	0.25
ESVI, ml/BSA^1.3^/year	0.53 (0.27)	1.39 (0.29)	**0.03**
Mass index, g/BSA^1.3^/year	− 1.36 (0.22)	− 1.07 (0.23)	0.36
EF %/year	− 0.24 (0.10)	− 0.61 (0.10)	**0.01**
Mass-to-volume ratio, /year	− 0.02 (0.003)	− 0.02 (0.003)	0.39
ESWS, kPa/year	0.30 (0.11)	0.26 (0.11)	0.78

Bold indicates p-value < 0.05

^a^All estimates significantly differed from zero, denoting significant change over time

^b^A p-value less than 0.05 indicates that the slopes of the two groups differ

## Discussion

This study demonstrated a progressive decline in CMR-derived measurements of ventricular performance in a cohort of older children and young adults with a Fontan circulation. In particular, EDVI increased by about 1 ml/BSA^1.3^ per year and EF (%) decreased by about 0.4 per year. Ventricular mass and mass-to-volume ratio decreased over time while ESWS increased. Those with the composite outcome of death, heart transplantation, or transplant listing demonstrated faster increase in EDVI. Patients with RV dominant hearts fared worse with faster decline in ventricular function and a higher rate of composite outcome.

Longitudinal studies using transthoracic echocardiography have shown progressive ventricular dilation and dysfunction after the Fontan operation; however, the clinical importance of these changes are not well known [16–19]. Prior publications from our institution using cross-sectional cohorts that overlap with the current study have demonstrated relationship between ventricular dilation and dysfunction with transplant-free survival [10, 12]. In the current study, over a median follow-up period of 10 years, 14% met the composite outcome and this study reaffirmed the association of this outcome with ventricular dilation and dysfunction. In addition, it revealed an association between rate of change of ventricular volume and the composite outcome. Those with the outcome had an estimated increase in EDVI of 4.7 ml/BSA^1.3^ per year compared to 0.8 ml/BSA^1.3^ in those without the outcome. Ventricular dilation may be attributable to several factors including volume loading lesions such as valvular regurgitation and aortopulmonary collaterals, as well as remodeling related to primary myocardial injury and dysfunction [20, 21]. In contrast to acquired myocardial disorders, the systemic single ventricle is exposed to abnormal hemodynamic stress beginning from prenatal life, which may predispose it to early failure. These findings suggest suboptimal ventricular remodeling in response to the hemodynamic perturbations in the Fontan circulation.

Patients with RV dominant functional single ventricles performed worse compared to those with LV dominance in the current study. This is not surprising as an LV would be expected to perform and remodel better as a functional single ventricle as compared to an RV which has evolved to support a low-pressure and low-resistance pulmonary circulation. More longitudinal myofiber orientation in the RV is also mechanically disadvantageous for pressure generation [22]. RV dominant ventricles showed a faster increase in ESVI of about 1.4 ml/BSA^1.3^/year compared to 0.5 ml/BSA^1.3^/year in LV dominant ventricles as well as a faster decline in EF (%) of 0.61/year compared to 0.24/year in the LV group. RV dominance was associated with a higher rate of composite outcome (21%) compared to LV (8%) a finding similar to a prior analysis of an overlapping cohort from our institution [15]. The underlying mechanisms for worse performance of the RV as a systemic pump may extend beyond the morphology and myofiber architecture. For example, the most common anatomic diagnosis with RV dominance is hypoplastic left heart syndrome and these patients are more likely to have had aortic arch reconstruction, aortopulmonary (Damus-Kaye-Stansel) anastomosis, higher number of surgical procedures, and longer cardiopulmonary bypass times which are factors that may influence long-term remodeling and performance of the ventricle.

### Limitations

The generalizability of these findings to the entire Fontan population may be limited due to a number of reasons. This is a CMR-based study and patients with pacemakers and implanted defibrillators were excluded. Only patients who had CMR studies without interim catheter or surgical interventions were included; hence, potentially sicker patients may have been excluded. The models used assumed linear changes over time; other patterns of change may be present. Associations of outcome with wall stress may differ if an alternative method of wall stress calculation were used. Effect of potential contributors to ventricular size such as aortopulmonary collateral flow, fenestrations or baffle leaks, venous collaterals, and LGE could not be evaluated in this study due to incomplete data on several initial CMRs. Despite these limitations, the study provides important overview of the trends in ventricular size and function in this population and insight into differences between LV and RV dominant hearts.

## Conclusions

Over a median follow-up period of 10 years, single ventricles in the Fontan circulation exhibited a steady decline in performance with an increase in EDVI, ESVI, and ESWS, and a decrease in EF, mass index, and mass-to-volume ratio. Patients with the composite outcome of death, heart transplantation, or transplant listing showed a faster increase in ventricular EDVI. Patients with rapid increase in EDVI (> 5 ml/BSA^1.3^/year) may be at a higher risk of adverse outcomes and may benefit from closer surveillance. Patients with dominant RVs are more likely to have the composite outcome and exhibit worse remodeling compared to dominant LVs.

## Supplementary Information

**Additional file 1.** Supplementary tables to support results.

## Data Availability

The datasets used and/or analyzed during the current study are available from the corresponding author on reasonable request.

## Abbreviations

AVVR: Atrioventricular valve regurgitation
BSA: Body surface area
CMR: Cardiovascular magnetic resonance
EDVI: End-diastolic volume index
EF: Ejection fraction
ESVI: End-systolic volume index
ESWS: End-systolic wall stress
IQR: Interquartile range
LGE: Late gadolinium enhancement
LV: Left ventricle/left ventricular
RV: Right ventricle/right ventricular
SD: Standard deviation
SE: Standard error
