# Supplementary material for: Longitudinal changes in ventricular size and function are associated with death and transplantation late after the Fontan operation
Source: J Cardiovasc Magn Reson. 2022 Nov 14;24:56. doi: 10.1186/s12968-022-00884-y (PMC9661807; doi:10.1186/s12968-022-00884-y)
Supplement: Supplementary file 1 — Additional file 1. Supplementary tables to support results. [file 12968_2022_884_MOESM1_ESM.docx]

**Supplement**

**Table 1A.** Model-based Estimates of Annual Change in CMR Parameters after Excluding Extreme Outliers (outside of IQR*3).

| Variables | N of CMRs | Estimated Annual Change (SE) | p-value |
| --- | --- | --- | --- |
| EDVI, ml/BSA^1.3^ | 487 | 0.94 (0.20) | **<.001** |
| ESVI, ml/BSA^1.3^ | 486 | 0.84 (0.14) | **<.001** |
| Mass index, g/BSA^1.3^ | 478 | -1.10 (0.15) | **<.001** |
| EF % | 489 | -0.41 (0.07) | **<.001** |
| Mass-to-volume ratio | 477 | -0.02 (0.002) | **<.001** |
| ESWS, kPa | 415 | 0.21 (0.07) | **0.004** |

EDVI, end-diastolic volume index; EF, ejection fraction; ESVI, end-systolic volume index; ESWS, end-systolic wall stress.

**Table 1B.** Model-based Estimates of Annual Change in CMR Parameters after Excluding Outliers (Outside of IQR*1.5).

| Variables | N of CMRs | Estimated Annual Change (SE) | p-value |
| --- | --- | --- | --- |
| EDVI, ml/BSA^1.3^ | 479 | 0.97 (0.19) | **<.001** |
| ESVI, ml/BSA^1.3^ | 475 | 0.80 (0.13) | **<.001** |
| Mass index, g/BSA^1.3^ | 467 | -0.92 (0.14) | **<.001** |
| EF % | 484 | -0.38 (0.07) | **<.001** |
| Mass-to-volume ratio | 455 | -0.01 (0.002) | **<.001** |
| ESWS, kPa | 407 | 0.25 (0.05) | **<.001** |

**Table 3A.** Estimates of Annual Change in CMR Parameters by Composite Outcome Status after Excluding Extreme Outliers (Outside of IQR*3).

| Variable | N | Composite outcome | No Composite outcome | p-value |
| --- | --- | --- | --- | --- |
| EDVI, ml/BSA^1.3^ | 487 | 3.45 (0.86) | 0.83 (0.20) | **0.003** |
| ESVI, ml/BSA^1.3^ | 486 | 2.63 (0.60) | 0.76 (0.14) | **0.002** |
| Mass index, g/BSA^1.3^ | 478 | -1.08 (0.64) | -1.05 (0.15) | 0.96 |
| EF % | 489 | -0.78 (0.31) | -0.39 (0.07) | 0.23 |
| Mass-to-volume ratio | 477 | -0.04 (0.01) | -0.02 (0.00) | **0.030** |
| ESWS, kPa | 415 | 0.69 (0.37) | 0.20 (0.07) | 0.19 |

**Table 3B.** Estimates of Annual Change in CMR Parameters by Composite Outcome Status after Excluding Outliers (Outside of IQR*1.5).

| Variable | N | Composite outcome | No Composite outcome | p-value |
| --- | --- | --- | --- | --- |
| EDVI, ml/BSA^1.3^ | 479 | 3.83 (0.84) | 0.83 (0.20) | **<.001** |
| ESVI, ml/BSA^1.3^ | 475 | 1.71 (0.57) | 0.76 (0.13) | 0.108 |
| Mass index, g/BSA^1.3^ | 467 | -0.10 (0.66) | -0.93 (0.14) | 0.220 |
| EF % | 484 | -0.43 (0.31) | -0.39 (0.07) | 0.884 |
| Mass-to-volume ratio | 455 | -0.013 (0.007) | -0.01 (0.002) | 0.771 |
| ESWS, kPa | 407 | 0.06 (0.35) | 0.26 (0.07) | 0.566 |

**Table 6.** Linear mixed models analysis for interaction between CMR parameters over time and age group

| Variable | N | Age ≤ 13 yrs | Age 13-20 yrs | Age > 20 yrs | p-value |
| --- | --- | --- | --- | --- | --- |
| EDVI, ml/BSA^1.3^ | 490 | 0.94 (0.40) | 0.86 (0.40) | 1.20 (0.43) | 0.837 |
| ESVI, ml/BSA^1.3^ | 490 | 1.10 (0.34) | 0.81 (0.34) | 0.86 (0.36) | 0.809 |
| Mass index, g/BSA^1.3^ | 481 | -1.07 (0.27) | -1.18 (0.27) | -1.44 (0.28) | 0.627 |
| EF % | 490 | -0.60 (0.12) | -0.38 (0.12) | -0.24 (0.13) | 0.120 |
| Mass-to-volume ratio | 481 | -0.02 (0.00) | -0.02 (0.00) | -0.03 (0.00) | 0.433 |
| ESWS, kPa | 417 | 0.27 (0.14) | 0.22 (0.13) | 0.33 (0.14) | 0.857 |

**Table 7.** Estimates of Annual Change in CMR Parameters by Presence of > Mild AVVR/AR

| Variable | N | >Mild AVVR/AR | ≤ Mild AVVR/AR/Unknown | p-value |
| --- | --- | --- | --- | --- |
| EDVI, ml/BSA^1.3^ | 490 | 0.62 (0.71) | 1.06 (0.25) | 0.559 |
| ESVI, ml/BSA^1.3^ | 490 | 0.73 (0.59) | 0.98 (0.21) | 0.699 |
| Mass index, g/BSA^1.3^ | 481 | -1.79 (0.47) | -1.15 (0.17) | 0.195 |
| EF % | 490 | -0.33 (0.21) | -0.43 (0.08) | 0.654 |
| Mass-to-volume ratio | 481 | -0.02 (0.01) | -0.02 (0.00) | 0.539 |
| ESWS, kPa | 417 | 0.23 (0.22) | 0.30 (0.08) | 0.761 |

**Table 8.** Estimates of Annual Change in CMR Parameters by Presence of a Significant Sized Secondary Ventricle

| Variable | N | Significant second ventricle present | Significant second ventricle absent | p-value |
| --- | --- | --- | --- | --- |
| EDVI, ml/BSA^1.3^ | 490 | 0.01 (0.43) | 1.41 (0.28) | **0.007** |
| ESVI, ml/BSA^1.3^ | 490 | 0.36 (0.36) | 1.17 (0.24) | 0.062 |
| Mass index, g/BSA^1.3^ | 481 | -1.61 (0.29) | -1.07 (0.19) | 0.117 |
| EF % | 490 | -0.32 (0.13) | -0.45 (0.09) | 0.387 |
| Mass-to-volume ratio | 481 | -0.02 (0.00) | -0.02 (0.00) | 0.393 |
| ESWS, kPa | 417 | 0.24 (0.14) | 0.29 (0.09) | 0.757 |

**Table 9.** Results of Interobserver Agreement Analysis for Ventricular End-diastolic Volume

| Variable | N  pair | Observation 1 | Observation 2 | Mean diff ± SD  (95% CI) | % error*  Median (IQR) | ICC  (95% CI) | CV, % |
| --- | --- | --- | --- | --- | --- | --- | --- |
| EDV (ml) | 20 | 169.5 ± 45.2 | 168.7 ± 45.3 | -0.8 ± 5.3 (-11.2, 9.5) | 2.6 ± 1.5 2.6 (1.3, 3.7) | 0.99 (0.98, 1.00) | 1.83 |

When two ventricles contributed to the systemic circulation, EDV represents the sum of both ventricular volumes.

**Figure 4.** Bland-Altman Plot Demonstrating Interobserver Agreement for Ventricular End-diastolic Volume

**
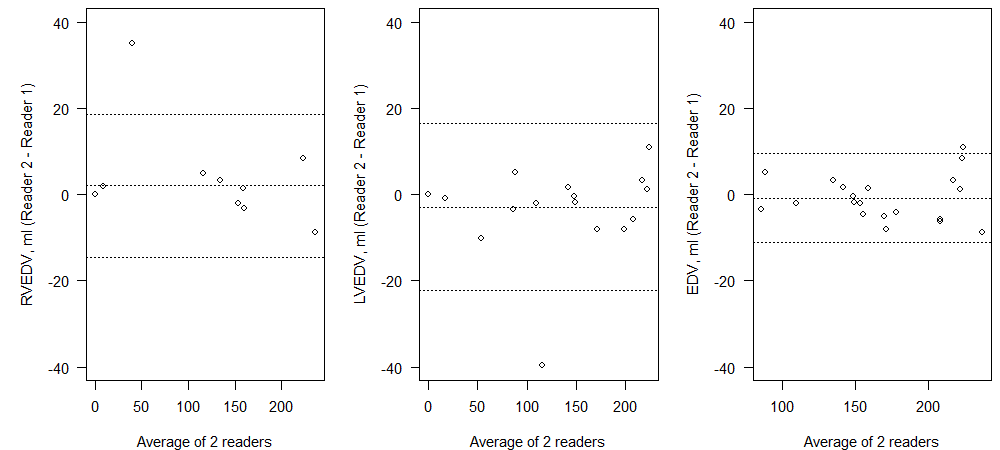
**
